# Global Burden of Cardiac Amyloidosis in Heart Failure: A Systematic Review and Meta-Analysis

**DOI:** 10.21203/rs.3.rs-8672903/v1

**Published:** 2026-01-23

**Authors:** Syed Bukhari, Mohammad Hamza, Muhammad Abdul Rehman, Claire Twose, Zubair Bashir

**Affiliations:** Johns Hopkins University

**Keywords:** Cardiac amyloidosis, global burden, transthyretin amyloidosis, heart failure, gender disparities

## Abstract

Cardiac amyloidosis (CA) is an infiltrative cardiomyopathy increasingly recognized as an important contributor to heart failure (HF), particularly with advances in noninvasive diagnostics. However, the global prevalence of CA remains poorly defined, with substantial variability across regions. We conducted a systematic review and meta-analysis of studies reporting the prevalence of CA among HF populations. The protocol was registered with PROSPERO and followed the PRISMA reporting guideline. Searches of PubMed Central, Cochrane, EMBASE and Web of Science identified eligible studies from inception through 2025. CA was diagnosed using technetium-labeled bone scintigraphy or biopsy. A random-effects proportional meta-analysis was conducted to estimate the pooled prevalence and assess geographic, subtype, sex-based, and study design–related differences. Twenty-eight studies encompassing 7,393 HF patients were included, of whom 627 were diagnosed with CA. The pooled prevalence of CA among HF patients was 10% (95% CI, 7%–13%), with substantial heterogeneity (prediction interval, 2–41%). Prevalence varied by region, ranging from 6% in North America to 15% in Asia, though subgroup differences were not statistically significant. Wild-type transthyretin amyloidosis (ATTRwt-CA) accounted for the majority of cases, representing 76% (95% CI, 57%–89%) of CA diagnoses and a pooled prevalence of 11% among screened populations. Approximately 24% of CA cases occurred in females. In conclusion, CA is present in one in ten patients with HF worldwide, with ATTRwt-CA being the predominant subtype. Uneven geographic distribution may suggest underdiagnosis, particularly in underrepresented regions and in females. Standardized, multinational studies are needed to define the global burden of CA better and guide equitable screening strategies.

## Introduction

Cardiac amyloidosis (CA) is an infiltrative cardiomyopathy caused by the extracellular deposition of misfolded amyloid fibrils within the myocardium, leading to progressive ventricular stiffness, arrhythmias, heart failure (HF), and increased mortality [1, 2]. The two predominant subtypes are light chain (AL-CA) and transthyretin (ATTR-CA) amyloidosis. Until recently, CA, particularly ATTR-CA, required invasive tissue biopsy and hence was underrecognized by clinicians and underdetected in the community. With advances in noninvasive diagnostic techniques, especially CA subtype differentiation with technetium-labeled bone scintigraphy, and comprehensive tissue characterization with cardiac magnetic resonance imaging, the epidemiology of CA has evolved in recent years [3]. Current guidelines now support a nonbiopsy diagnostic pathway for most patients with ATTR-CA amyloidosis, enabling broader recognition of the disease across clinical settings [4]. However, despite these advances, CA remains widely underdiagnosed and misdiagnosed, and a significant proportion of affected individuals are still identified incidentally at autopsy [5, 6].

With more accessible diagnostic strategies and heightened clinical awareness, CA, particularly ATTR-CA, has emerged as a more common contributor to HF than previously appreciated[7]. Initial epidemiologic studies came predominantly from Europe and North America, where ATTR-CA was reported in older adults with heart failure with preserved ejection fraction (HFpEF) or unexplained left ventricular (LV) hypertrophy [8, 9]. Subsequent studies from other regions have similarly identified ATTR-CA in diverse populations, suggesting that the condition is globally distributed rather than confined to specific ancestry groups [10, 11]. However, estimates vary widely between countries and healthcare systems, reflecting differences in diagnostic availability, referral pathways, and patient selection.

It remains unclear whether true variations in prevalence exist worldwide or whether observed differences reflect inconsistent detection. Although contemporary reviews often highlight the rising recognition of CA globally, no comprehensive assessment of its worldwide prevalence has been performed. Given the expanding availability of disease-modifying therapies for CA and the growing recognition of underdiagnosis across many regions, a rigorous synthesis of global epidemiologic data is needed. We therefore conducted a systematic review and meta-analysis of published studies on the prevalence of CA to estimate its pooled prevalence and to examine geographic variation in disease burden globally.

### Methodology and Analysis

The protocol of this systematic review and meta-analysis was registered with the International Prospective Register of Systematic Reviews (PROSPERO) in July 2024 (Registration number: CRD420251108781). This review is reported in accordance with the published Preferred Reporting for Systematic Review and Meta-analysis guidelines (PRISMA).

### Search Strategy

A health sciences librarian developed a comprehensive search strategy (Appendix A) to capture articles that fit the eligibility criteria for this review. The search strategy included a mix of keywords and controlled vocabulary terms for the concepts of “Cardiac Amyloidosis” and “Heart Failure” and was run in the following academic research databases on August 06, 2025: PubMed, and August 14, 2025, for: Web of Science, EMBASE, Cochrane, and CENTRAL. These databases were searched from inception to the date of the search. We used a snowball approach to search for references of the studies whose full texts were reviewed. All results from the search were uploaded to COVIDENCE.

### Inclusion/Exclusion Criteria

We included studies that reported epidemiological data on CA in HF patients. This included the prevalence of CA in HF patients. Those with populations under 18 years of age, pregnant patients, case reports/series, and/or those with fewer than 30 patients were excluded. For this review, only studies that evaluated patients with HF and CA, diagnosed by either Tc-pyrophosphate scintigraphy (ATTR-CA) or biopsy (ATTR-CA or AL-CA) were included.

### Screening/Data Extraction

**Figure 1:**
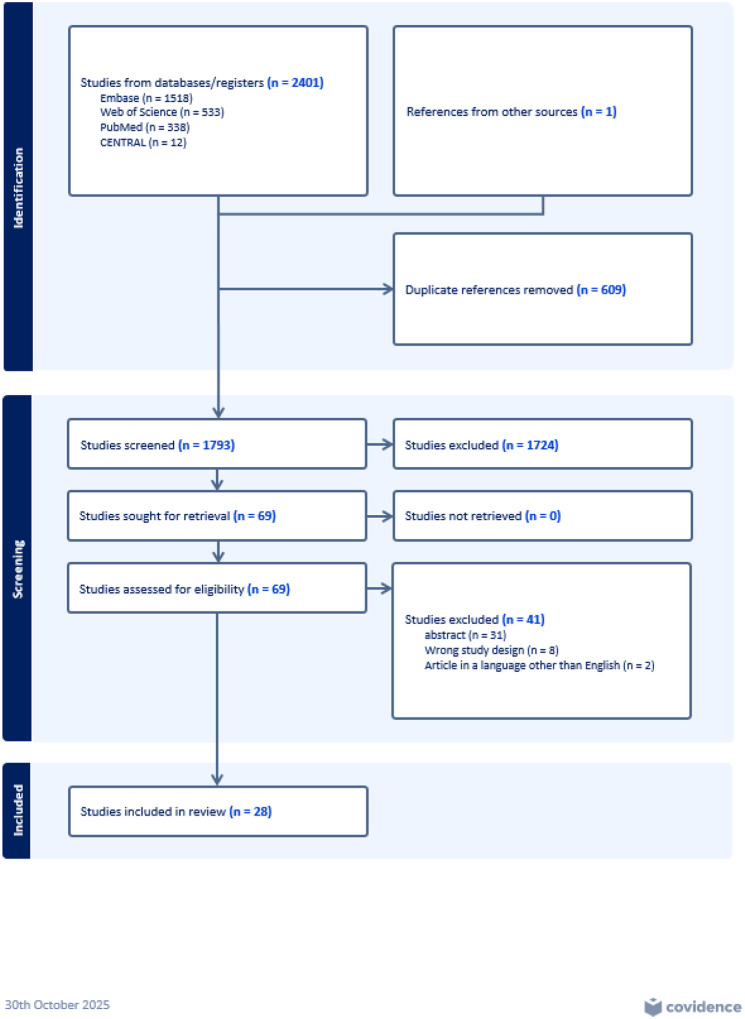
Prisma Flow Diagram

COVIDENCE software was used to screen studies and remove duplicates. Subsequently, the articles were screened independently by two reviewers (SB and ZB). They reviewed the full text of each article based on the pre-defined inclusion and exclusion criteria. Any conflicts were resolved by discussion between the two reviewers. In case a mutual consensus failed to lead to a decision, a third author was consulted, who reviewed the study and made the decision. Articles were first screened based on abstract and study titles. Shortlisted articles underwent a full-text review. The full search methodology is presented in Fig. 1.

The data was extracted from the selected articles onto a Microsoft Excel spreadsheet. For each study, the following information was retrieved: first author, year of publication, country, study design, total number of patients included in the study, number of patients with CA, number of patients stratified by CA subtypes (if available), number of males, etc.

### Outcomes

The primary outcome of this study was the pooled prevalence of CA in HF patients across the existing literature. Secondary outcomes included pooled estimates of CA subtypes, regional differences in prevalence across CA and its subtypes, sex-related variation in CA prevalence, and prevalence stratified according to study design.

### Risk of Bias

Risk of bias was assessed using the Joanna Briggs Institute (JBI) Critical Appraisal Checklist for Studies Reporting Prevalence Data[12]. Each study was evaluated across nine items addressing the appropriateness of the sampling frame, sampling method, and sample size; the description of study subjects and setting; the validity and reliability of the measurement methods; and the adequacy of statistical analysis and response rate. Each item was rated as “Yes,” “No,” or “Not applicable.”

An overall risk of bias judgment was then assigned based on the pattern of responses across the nine items. Studies were classified as having low risk of bias if all items were rated “Yes,” or if only one non-critical item was rated “No.” Studies were classified as having a moderate risk of bias if two or more non-critical items were rated “No,” or if any critical item was rated “No.” High risk of bias was assigned when three or more non-critical items were rated “No,” or when two or more critical items were rated “No.” Critical items included questions 1, 2, 5, and 6, which evaluate the sampling frame, sampling method, validity of measurement, and standardization of measurement, respectively.

### Statistical Analysis

We conducted a proportional meta-analysis using a logit transformation under random-effect models to account for inter-study differences according to the Maximum Likelihood method. The I^2^ characteristic reflects the observed variance between studies that cannot be explained by sampling error[13]. We assessed publication bias visually using funnel plot asymmetry and numerically through Begg’s rank test and Egger’s regression test. Prediction intervals reflect the heterogeneity or range of predicted estimates for each outcome[14]. A sensitivity analysis was conducted using the leave-one-out method. A *p*-value < 0.05 was considered statistically significant.

All analysis, forest plots, and geographical maps in this study were conducted or prepared using R (version 4.4.0) and RStudio using the libraries ggplot2 and maps. All meta-analyses were performed using the package meta.

## Results

The initial search using a comprehensive search strategy revealed a total of 2,401 articles. 1,518 articles were identified on EMBASE, 533 on Web of Science, 338 on PubMed, and 12 on CENTRAL. After removing 609 duplicates, 1,793 articles remained for screening. The abstracts of these were reviewed, and 70 articles remained after filtering those that did not meet the inclusion criteria. The full texts of 69 articles were independently assessed by two reviewers (SB, ZB) against the prespecified inclusion and exclusion criteria. Ultimately, 28 studies met the eligibility criteria and were included in this review.

### Risk of Bias in the included studies

Most studies were assessed as having a low risk of bias based on unclear criteria for determining sample size[8, 15–32]). Seven studies were judged to have a moderate risk of bias: six failed to meet one critical item, and one study had two non-critical items rated as “No.”[9–11, 33–36]. Two studies were classified as having a high risk of bias[37, 38]. One of these did not satisfy two critical items, while the other had three non-critical items and one critical item rated as “No.” (Table 1).

### Study Characteristics

A total of 28 studies were included, encompassing 7,393 patients evaluated for CA. Of these, 627 patients (approximately 8.4%) were diagnosed with CA across differing clinical settings, diagnostic strategies, and phenotypes [8–11, 15–38]. Studies varied widely in design, including prospective (n = 21) and retrospective (n = 7) cohorts, with both single-center (n = 18) and multi-center (n = 10) studies represented. The studies spanned 14 countries, with the largest contributions from the USA, Spain, and Japan (Table 2).

### Patient Demographics

Across all studies, the mean/median age of enrolled populations ranged from 66 to 88 years, with most cohorts reporting a predominantly older population typical of amyloid cardiomyopathy. Sex-based prevalence of CA was reported in most cohorts, indicating that CA-positive patients were predominantly male. Most studies reporting HF phenotype had most patients with preserved HF (left ventricular ejection fraction ≥ 50%) (Table 2).

### Studies Reporting Prevalence of Cardiac Amyloidosis

The prevalence of CA within individual studies varied substantially, ranging from 0.5% to 31%, reflecting differences in inclusion criteria, case mix, and diagnostic criteria. Lowest prevalence was reported by Chang *et al*. (0.5%, AL-CA only), while the highest prevalence was reported by Bennani *et al*. (30.6%). Moreover, CA subtype reporting was available in 18 of 28 studies. Wild-type ATTR-CA (ATTRwt-CA) represented most typed cases, with prevalence ranging from 7–20% among tested population

Variant ATTR-CA (ATTRv-CA) was rare, with only small counts reported (typically < 10 per study), often identified in regions with endemic mutations (e.g., Spain, Japan). AL-CA was variably represented (0–36 cases per study), more commonly identified in biopsy-driven cohorts, with prevalence clustering between 5–20% in HFpEF or LVH-based screening populations.

A few studies reported large discrepancies between ATTR-CA and AL-CA distributions depending on screening methods (e.g., bone scintigraphy vs. biopsy vs. mixed modalities). Notably, Chang *et al*. (AL-CA only screening cohort) identified only 6 AL-CA cases among 1173 patients, indicating a very low prevalence when testing broad HF referral populations (Table 2).

### Outcome assessment

**Figure 2:**
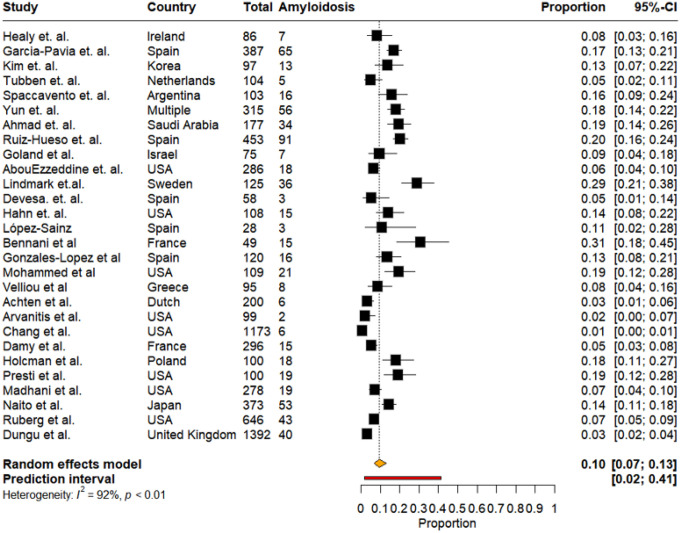
Pooled prevalence of cardiac amyloidosis across all studies.

Across the 28 studies included in the primary meta-analysis, the pooled prevalence of CA was 10% (95% CI, 7%–13%, I^2^: 92%) with a prediction interval ranging from 2% to 41%, based on a random-effects model. However, a sensitivity analysis did not reveal any one study causing a clinically significant change in the pooled prevalence (Supplementary Table 1). The largest absolute difference in prevalence was 1.1% after omitting the study by Chang *et al*. Prevalence estimates varied substantially across studies, ranging from 1% to 31%, with the highest estimate reported by Bennani *et al*. and the lowest by Chang *et al*. (Fig. 2).

### Subgroup analysis

**Figure 3:**
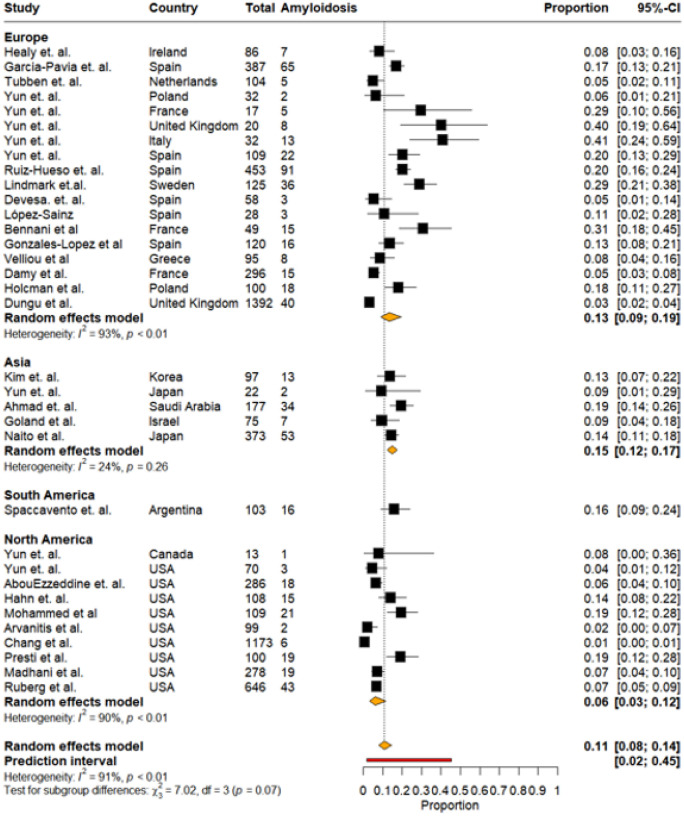
Pooled prevalence of cardiac amyloidosis stratified by geographical regions

**Figure 4:**
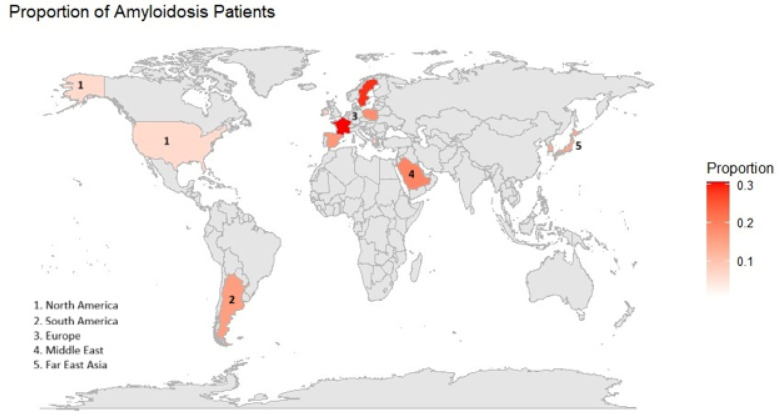
Heat map showing regional prevalence of CA

Regional Distribution: The overall pooled prevalence of CA, stratified by geographic regions—Europe, Asia, South America, and North America —was 11% (95% CI: 8%–14%, I^2^:91%), with a wide prediction interval ranging from 2% to 45% (Fig. 3,4).

Europe included the largest number of studies. Reported prevalence ranged widely across countries, but the pooled estimate stabilized at 13% (95% CI: 9%–19%, I^2^:93%). Asia demonstrated the narrowest prediction interval, ranging from 11% to 19%. Individual study estimates showed moderate variation, and the regional pooled prevalence was 15% (95% CI: 12%–17%).

South America was represented by a single study from Argentina, estimating a prevalence of 16% (95% CI: 9%–24%, I^2^:NA).

North America showed a wide prediction interval ranging from 0% to 47%, with individual study estimates ranging from near zero to 19%. Exclusion of data from the Canadian study did not significantly affect the prediction interval. The regional pooled prevalence was lower than other regions at 6% (95% CI: 3%–12%, I^2^:90%). No significant subgroup differences (p = 0.07) in the regional variation of CA prevalence were noted (Fig. 3).

**Figure 5:**
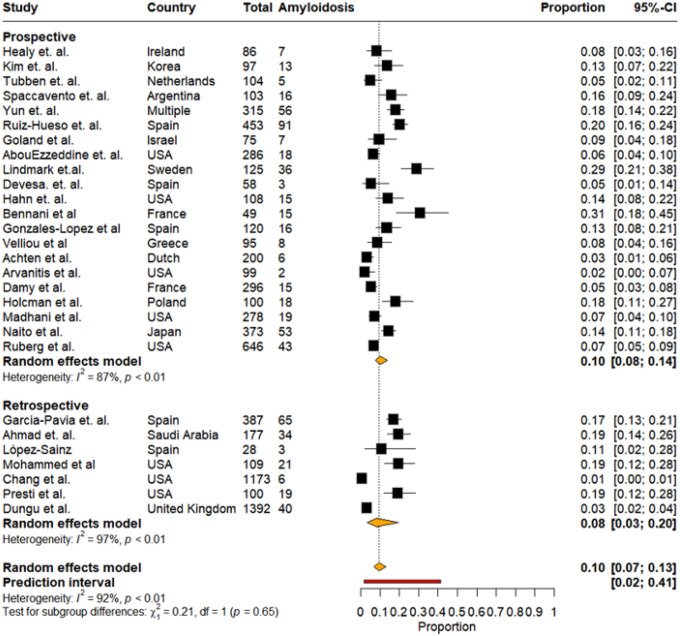
Pooled prevalence of cardiac amyloidosis stratified by study design (prospective vs retrospective)

Study Design: After stratification by study design, 21 studies constituted prospective cohorts, while 7 were part of retrospective cohorts. Among prospective studies, the pooled prevalence of CA was 10% (95% CI: 8%−14%, I^2^:87%). Prevalence estimates ranged widely—from 2% to 31%—reflecting variations in study populations, diagnostic criteria, and geographic representation. Retrospective studies demonstrated a pooled prevalence of 8% (95% CI: 3%–20%, I^2^:92%). Individual estimates varied from 1% to 19%, with larger cohorts generally showing narrower confidence intervals (Fig. 5). However, the difference in pooled prevalence between both study designs was not significant (p = 0.65).

**Figure 6:**
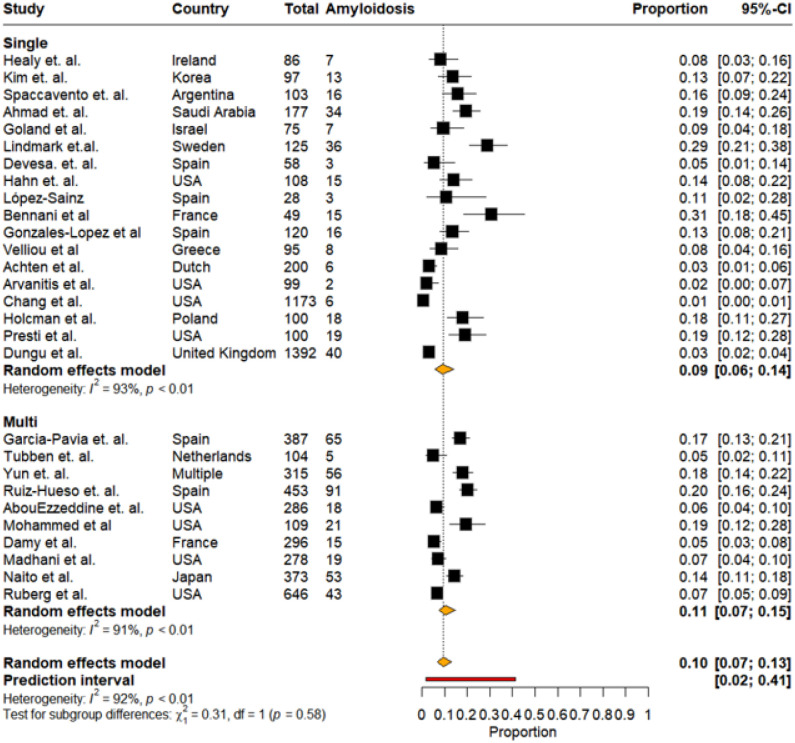
Pooled prevalence of cardiac amyloidosis stratified by center (single vs multicenter)

The studies were also stratified as single (n = 18) vs. multicenter (n = 10) cohorts. Among single-center studies, the pooled prevalence of CA was 9% (95% CI: 6%–14%, I^2^:93%), Individual estimates varied widely, ranging from 1% to 31%. Multi-center studies demonstrated a comparable pooled prevalence of 11% (95% CI: 7%–15%, I^2^:91%). Prevalence estimates ranged from 5% to 20%, with larger sample sizes contributing to narrower confidence intervals in several cohorts and ultimately a higher, I^2^ value (Fig. 6).

**Figure 7:**
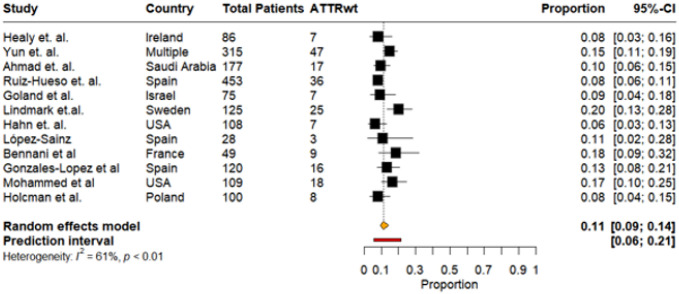
Pooled prevalence of ATTRwt among total patients in studies that reported CA subtypes, ATTRwt and AL

CA Subtypes: Among HF studies reporting CA subtypes, 12 studies provided data on both ATTRwt-CA and AL-CA. Across these cohorts, the pooled prevalence of ATTRwt-CA among all evaluated patients was 11% (95% CI: 9%–14%, I^2^: 61%), with a narrow prediction interval ranging from 6% to 21%. Individual study estimates ranged from 8% to 20%, reflecting variation in population demographics, diagnostic strategies, and referral patterns. (Fig. 7). A sensitivity analysis revealed no significant effect of any one study on the overall pooled prevalence (**Supplementary Table 2**). The largest effect was carried by the study by Lindmark *et al*. causing an absolute difference in prevalence of just 0.7%.

**Figure 8:**
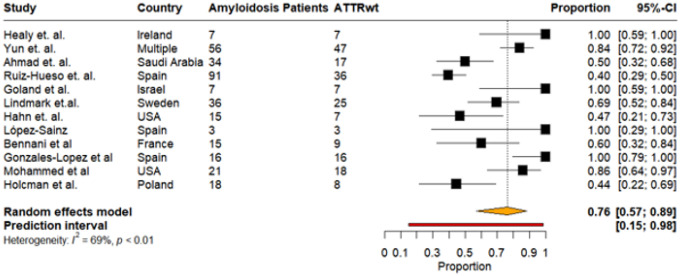
Pooled prevalence of ATTRwt among cardiac amyloidosis patients in studies that reported CA subtypes,ATTRwt and AL.

Among HF studies reporting CA subtypes, 12 studies provided data specifically on ATTRwt-CA. Across these cohorts, the pooled prevalence of ATTRwt-CA among all HF patients with CA was 76% (95% CI: 57%–89%, I^2^: 69%), with a wide prediction interval ranging from 15% to 98%. Individual study estimates ranged from 40% to 100%. (Fig. 8)

**Figure9:**
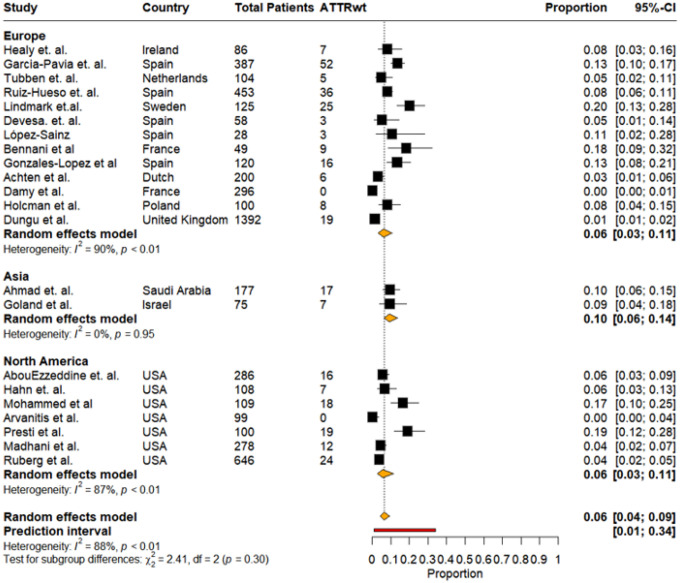
Pooled prevalence of ATTRwt among total patients in studies stratified by regions

Overall: The overall pooled prevalence of ATTRwt-CA among HF patients, stratified by geographic regions—Europe, Asia, South America, and North America —was 6% (95% CI: 4%–9%,I^2^: 88%), with a prediction interval ranging from 1% to 34% (Fig. 9). Europe included the largest number of studies. Pooled prevalence of ATTRwt-CA in Asia was 10% (95% CI: 6%–14%, I^2^: 0%). North America showed variable individual study estimates ranging from near zero to 19%. The regional pooled prevalence was slightly lower than Asia but similar to Europe at 6% (95% CI: 3%–11%). No significant subgroup differences (p = 0.30) in the regional variation of ATTRwt-CA prevalence were noted (Fig. 9).

**Figure 10:**
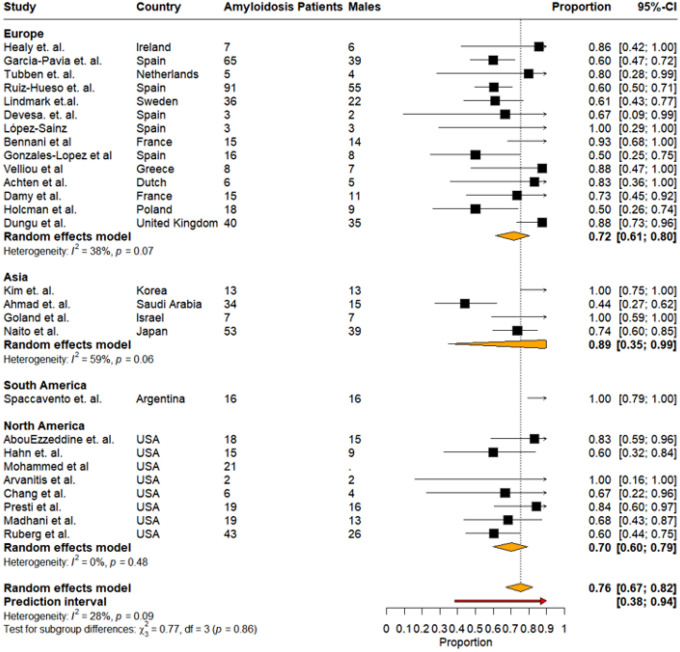
Pooled prevalence of cardiac amyloidosis among males stratified by regions.

The overall prevalence of males among HF patients with CA was 76% (95% CI: 67%–82%). Subgroup analysis by regions revealed that pooled prevalence of males was highest in Europe (n = 14), i.e.,72% (95% CI: 61%–80%, I^2^: 38%), with a wide-ranging prediction interval ranging from 38% to 94%, followed by Asia (4 studies) 89% (95% CI: 35%–99%, I^2^: 59%), South America (n = 1) 100% (95% CI: 79%–100%), and the lowest prevalence of males was noted in North America (n = 8), i.e., 70% (95% CI: 60%–79%, I^2^: 0%) (Fig. 10).

### Publication Bias Assessment

Funnel plot asymmetry was noted visually for the included studies, consistent with significant publication bias between the studies (Egger’s: p = 0.0096) (**Supplementary Fig. 1**).

## Discussion

### Global Prevalence and Geographic Variability

Our study demonstrates that the pooled prevalence of CA among patients with HF is approximately 10%. However, the available evidence is marked by substantial geographic imbalance. Most included studies originated from Europe and North America, with fewer contributions from Asia, the Middle East, and South America—and none from Africa. Several factors likely contribute to the paucity of data in African settings, including limited diagnostic infrastructure, low clinician awareness, and the absence of population-level surveillance systems [39]. Given the region’s disproportionate burden of HF–related mortality—some of which may be attributable to unrecognized ATTR-CA—these gaps have meaningful clinical and public health implications. This “data desert” limits our ability to accurately characterize global prevalence and may obscure important regional differences in diagnostic practices, population risk profiles, and healthcare access. Additionally, the higher prevalence observed in European cohorts compared with North American cohorts may reflect broader adoption of advanced imaging protocols, more systematic screening of at-risk populations, or differences in study design and referral patterns [40]. Alternatively, these differences could indicate true epidemiologic variation, although current data remain insufficient to draw definitive conclusions.

### Shifting Landscape of CA Subtypes

Our findings also reflect a notable shift in the epidemiology of CA. Across included studies, ATTR-CA—particularly ATTRwt-CA —was the predominant form of CA, whereas AL-CA, historically viewed as the most common CA subtype [41, 42], accounted for a smaller proportion of cases. This shift likely reflects the rapid evolution of diagnostic pathways for ATTR-CA, including the widespread use of bone scintigraphy and noninvasive diagnostic algorithms that enable prompt recognition of ATTR-CA without biopsy[3, 43]. Growing awareness among clinicians, along with increased screening in older adults and patients with unexplained LV hypertrophy or HFpEF, is potentially contributing to higher detection rates. Conversely, AL-CA remains dependent on timely hematologic evaluation and histological confirmation, and its lower reported prevalence in contemporary HF cohorts may reflect both true epidemiologic differences and potential under-recognition outside specialized centers. The markedly lower prevalence of ATTRv-CA compared with ATTRwt-CA reflects both the overall rarity of pathogenic TTR variants in the general population and the geographic concentration of endemic mutations.

### Sex Differences and Emerging Recognition in Females

Importantly, approximately 20% of identified ATTRwt-CA cases occurred in females. This challenges the traditional perception that ATTRwt-CA is an almost exclusively male disease, as earlier studies reported that 90–95% of ATTRwt-CA cases occurred in males.[44, 45]. While ATTR-CA, particularly ATTRwt-CA, remains more common in males, the increased proportion of female population likely reflects historical under-recognition and improved detection in the contemporary era, driven by greater disease awareness and more widely available noninvasive testing. Several sex-specific phenotypic differences may partly explain why ATTRwt-CA has historically been under-recognized in females despite a meaningful disease burden. Females generally exhibit lower absolute LV wall thickness, smaller body size–indexed parameters, and distinct patterns of cardiac remodeling compared with males. Consequently, reliance on non–body size–indexed wall thickness cutoffs potentially contributes to under-appreciation of ATTR-CA in affected females in the clinical settings. Additionally, symptoms of ATTR-CA often overlap with hypertensive heart disease or HFpEF[46], which may contribute to delayed recognition—particularly in female patients, who are less likely to fit the historically recognized male-predominant phenotype. Future studies are needed to delineate sex-specific phenotypes and evaluate whether revised diagnostic thresholds or targeted screening strategies could improve detection in females.

### Implications and Future Directions

Taken together, these findings highlight important gaps in the global understanding of CA. The observed prevalence of 10% among heart failure patients underscores the need for broader screening and improved awareness across diverse clinical settings. The geographic imbalance in existing data suggests that large segments of the world remain unstudied, raising concerns that regional differences in recognition and access to diagnostic modalities may contribute to underdiagnosis. The predominance of ATTR—and particularly ATTRwt-CA—calls for continued emphasis on early detection, given the growing availability of disease-modifying therapies that are most effective when initiated early. The emerging recognition of CA in females demands focused investigation to ensure equitable diagnosis and treatment. Finally, recognition of CA in heart failure patients may also influence treatment in this population, such as goal-directed medical therapy, especially in older patients which are more likely to be diagnosed with CA and HF[47]. Addressing these gaps through multinational, standardized studies will be essential to defining the true global burden of CA and optimizing care for affected patients.

### Limitations

This review has several important limitations. The high I^2^ reflects variance not attributable to sampling error across included studies. However, one should note that high I^2^ values are common in prevalence studies with continuous outcomes.[48, 49] In contrast, we encountered wide-ranging prediction intervals that indicate heterogeneity across studies. This was largely seen in studies from North America and Europe, but less in Asian studies. Similarly, we also encountered publication bias, which further affects the generalizability of our results. Readers should also consider the temporal and geographic variability across studies. Although a region-wise subgroup analysis was conducted, the temporal variability is more difficult to address, as prevalence is likely to change over the years during which the studies were conducted.

## Conclusions

Our study showed that CA is present in approximately 10% of patients with HF, with ATTRwt-CA as the predominant subtype. Although regional differences were not statistically significant, substantial heterogeneity and uneven global representation highlight persistent gaps in detection, including in female patients. These findings support broader, standardized screening and the need for multinational studies to better define the global burden of CA.

## Supplementary Material

Supplementary Files

This is a list of supplementary files associated with this preprint. Click to download.

- Supplementarysection.docx
- Table12.docx
